# Multi-omic analysis of canine aging uncovers conserved aging pathways

**DOI:** 10.1007/s11357-025-02029-2

**Published:** 2025-12-17

**Authors:** Zane Koch, Jessica L. Graves, Steve Annan, Phillip A. Frankino, Michelle Nelson, James E. McMahon, Ashley P. Tovar, Hubert C. Chen, Celine-Lea Halioua-Haubold, Matthew Peloquin

**Affiliations:** 1 Loyal Animal Health, Inc., Dallas, TX USA; 2 Program in Bioinformatics and Systems Biology, University of California San Diego, La Jolla, CA 92093 USA

**Keywords:** Canine aging, Longevity, Aging, Proteomics, Transcriptomics

## Abstract

Aging is a complex biological process characterized by molecular changes across multiple biological scales. While these alterations have been extensively studied in humans and rodents, the molecular changes associated with aging in dogs remain underexplored despite their relevance as a model for human aging. In this study, we profiled gene expression (*n* = 16,273 genes) and protein abundance (*n* = 2041 proteins) in whole blood and blood plasma from 40 laboratory beagles across young (3–5 years old, *n* = 10), old (8–9 years old, *n* = 17), and geriatric (10–14 years old, *n* = 13) life stages. We identified 816 genes and 40 proteins that significantly changed in abundance during aging, converging on pathways involved in DNA repair, collagen processing, and inflammation. Notably, these canine aging signatures overlapped with human age-associated genes, including those tied to the hallmarks of aging, reinforcing the existence of conserved aging mechanisms across species.

**Supplementary Information:**

The online version contains supplementary material available at https://doi.org/10.1007/s11357-025-02029-2.

## Introduction

Aging is a multifaceted biological process marked by a progressive decline in physiological function and an increased susceptibility to age-related diseases. It is the greatest risk factor for death from most diseases [8, 24], and as the world population ages, investigation and treatment of the aging process are of increasing importance [6]. While the molecular hallmarks of aging, such as genomic instability, epigenetic alterations, and mitochondrial dysfunction, have been extensively studied in model organisms like yeast, worms, flies, and mice [30], the progression of age-related molecular changes in companion dogs (*Canis lupus familiaris*) remains relatively unexplored.

Dogs, as companion animals, share many similarities with humans in terms of their life trajectory, environment, and susceptibility to age-related diseases—making them a valuable model for studying human aging. For instance, dogs naturally develop age-related morbidities, including cancer, metabolic disease, and neurodegenerative disorders, with similar incidence and progression to humans [21]. Due to their common environment, dogs and their owners tend to share allergies [28], can impact one another’s microbiota [52], and experience similar pollutants and pathogens [32]—suggesting that environmental impacts on the human aging process are likely to be reflected in dogs. The laboratory beagles, while housed in controlled conditions less resembling the human environment, have nonetheless been found to be highly relevant models of human disease [36]. Additionally, genetic studies of the determinants of dog lifespan across breeds have uncovered variants also associated with lifespan in humans [26, 41], such as in the *IGF1* gene [34]. These parallels have led to the use of dogs in testing putative lifespan-extending interventions, including caloric restriction [25] and rapamycin [5, 51], as well as treatments for age-related diseases such as Alzheimer’s disease [11].

While high-throughput genomic and proteomic studies have identified molecular signatures of aging in humans and model organisms [29, 37, 50], relatively few large-scale -omics studies have been conducted in dogs. A small number of transcriptomic studies have examined aging in dogs [23, 44], Kim, Jang, and Kim 2023), but have typically focused on compiling atlases of organ-specific transcriptional activity [2, 55, 56] or were limited to young and middle-aged dogs (< 9 years old) [54]. Likewise, age-associated alterations in plasma protein abundance have been studied in only a small subset of the canine proteome (< 30 proteins) [27, 38]. The most comprehensive molecular investigations of dog aging to date have instead focused on metabolomic and epigenetic data [19, 22, 53].

To advance our understanding of the molecular underpinnings of canine aging, we conducted a comprehensive multi-omic analysis of aging in laboratory beagles. By profiling gene expression and protein abundance in whole blood and blood plasma of 40 laboratory beagles, we identify molecular markers of aging in dogs and compare these to those observed in humans. Our study extends previous work by providing a thorough analysis of age-related changes in the canine transcriptome and proteome, with a particular emphasis on geriatric dogs (10–14 years old). Additionally, we make these data publicly available at https://loyal-for-stats.shinyapps.io/beagle-omics/, thereby facilitating further research into both dog and human aging.

## Results

### Comprehensive multi-omic profiling of laboratory beagles

**Fig. 1:**
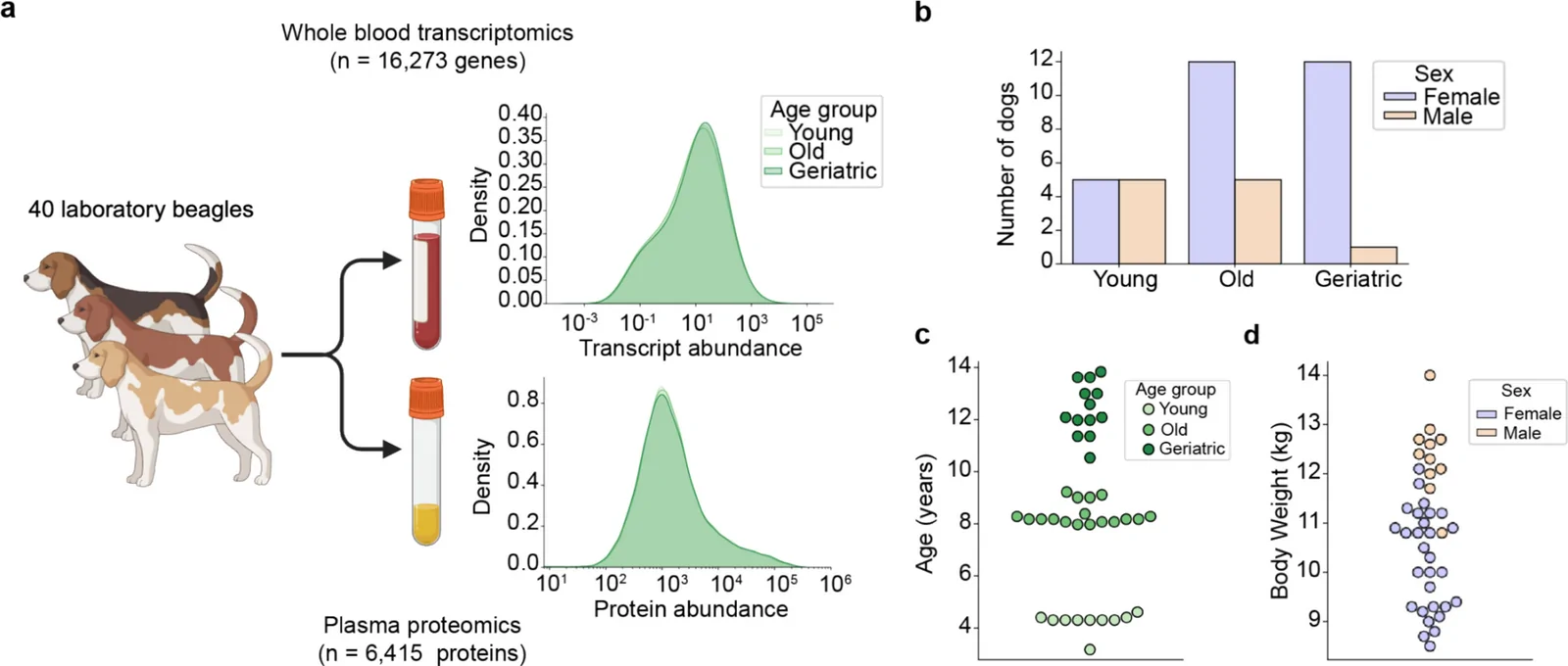
Study design and demographic overview of the laboratory beagle cohort. **a** Schematic representation of the multi-omic profiling study. Whole blood was collected from 40 laboratory beagles for RNA-sequencing (16,273 genes), and blood plasma was used for proteomic analysis (6415 proteins) via the SomaScan 7 K platform. **b** Bar chart illustrating the number of female and male dogs within each age group: young (3–5 years), old (8–9 years), and geriatric (10–14 years). **c** Dot plot showing the age distribution for all 40 dogs, colored by their assigned age group. Each point corresponds to the chronological age of a single dog. **d** Dot plot of individual dog weights (in kilograms), colored by sex. Each point represents the body weight of a single dog at the time of blood sample collection

To capture the molecular landscape of aging in dogs, we collected whole blood and plasma samples from 40 laboratory beagles spanning 3.3 to 13.7 years of age (Table 1). From the whole blood, we generated RNA-seq data capturing the expression of 16,273 genes, and from the plasma, we used the SomaScan 7 K platform to measure the abundance of 6,415 proteins (Fig. 1a, Methods). The wide age range (Fig. 1b, c) enabled the identification of age-related molecular changes across nearly the entire dog’s lifespan.

**Table 1:** Demographics of the laboratory beagle population

Characteristic	Overall (*n* = 40)	Young (*n* = 10)	Old (*n* = 17)	Geriatric (*n* = 13)
Age				
Mean (SD)	8.65 (3.10)	4.34 (0.38)	8.39 (0.41)	12.30 (0.97)
Range	3.30–13.70	3.30–4.70	8.00—9.20	10.50–13.70
Body weight (kg)				
Mean (SD)	10.82 (1.36)	11.24 (1.47)	10.95 (1.41)	10.32 (1.14)
Range	8.50–14.00	9.20–14.00	9.00–12.90	8.50–12.00
Sex				
F	29 (73%)	5 (50%)	12 (71%)	12 (92%)
M	11 (27%)	5 (50%)	5 (29%)	1 (7.7%)

Beagle dogs are typically fully grown by one year of age [20] have a median lifespan of approximately 16 years (Urfer et al. 2020) [1]. For subsequent analyses, we categorized the dogs into three age groups: young (3–5 years, *n* = 10), old (8–9 years, *n* = 17), and geriatric (10–14 years, *n* = 13). While all dogs in this cohort had reached sexual and developmental maturity, we use “young” to denote dogs in early adulthood with minimal age-related molecular changes. On average, the cohort weighed 10.82 ± 1.36 kg (mean ± stdev), with males generally weighing more than females (male = 12.38 ± 0.80 kg, female = 10.22 ± 1.01 kg; Fig. 1d, Table 1).

### Transcriptomic changes in metabolism, DNA repair, and collagen pathways with age

**Fig. 2:**
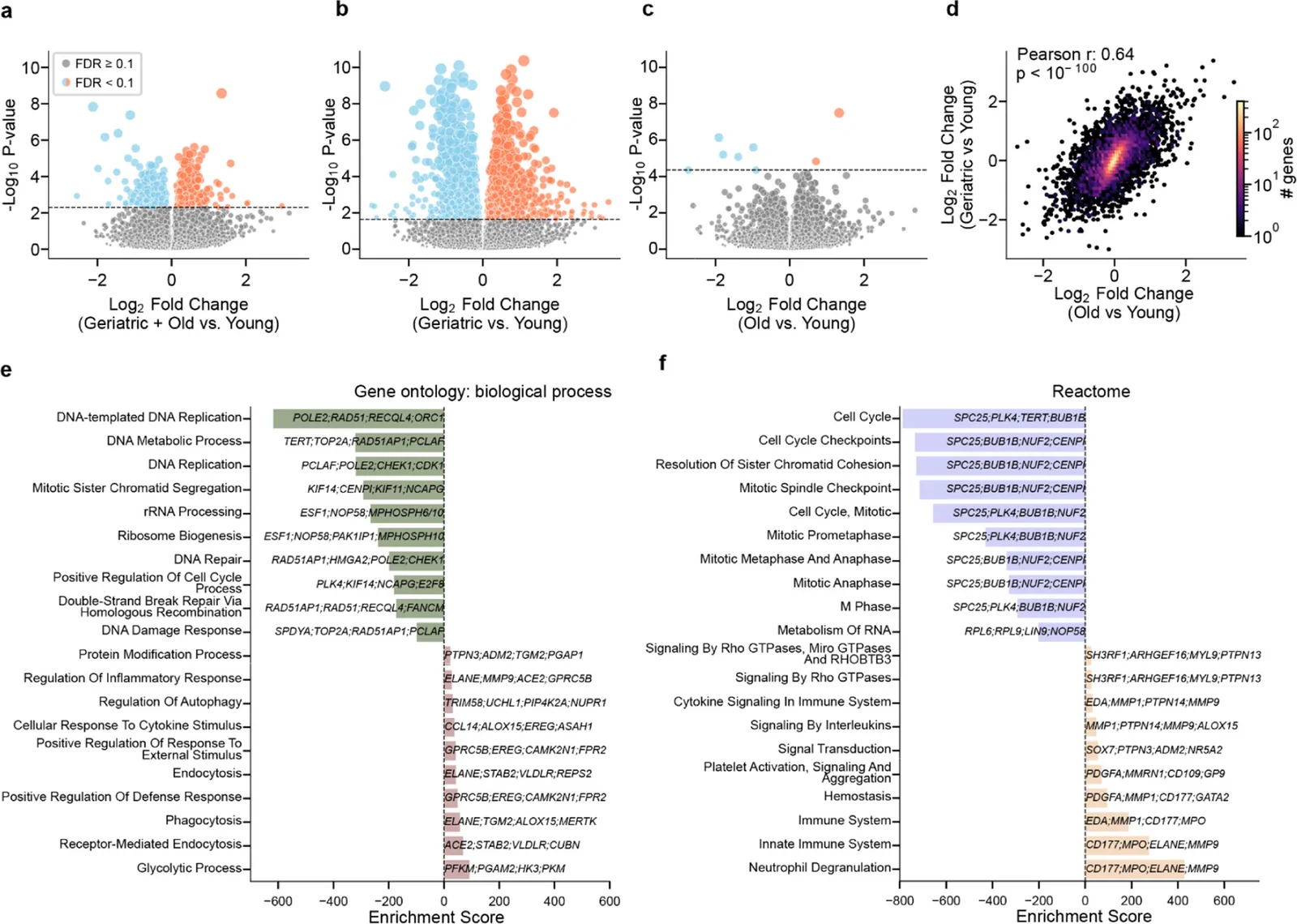
Transcriptomic alterations associated with aging in dogs. **a–c** Volcano plots depicting log_2_ fold change (*x*-axis) versus –log_10_ *p*-value (*y*-axis) for all detected genes (*n* = 16,273 genes) across three comparisons: **a** geriatric and old vs. young dogs, **b** geriatric vs. young dogs, and **c** old vs. young dogs. Genes with FDR-corrected *p*-values < 0.1 (blue/orange) are considered significantly altered, whereas those with FDR-corrected *p*-values ≥ 0.1 (gray) are not (Methods). Significance was assessed using two-sided Wald tests followed by FDR correction in DESeq2. **d** Scatter plot illustrating the log_2_ fold change of each gene in geriatric vs. young (*y*-axis) and old vs. young (*x*-axis) comparisons. Points are colored by their log-scaled density. **e** Gene-set enrichment analysis (GSEA) results based on GO pathways. The ten most significantly up- and down-regulated pathways are presented. Bar length (*x*-axis) indicates the enrichment score for genes downregulated (blue) or upregulated (red) with age in aged (geriatric and old) vs. young dogs (*n* = 30 dogs). **f** GSEA results for Reactome pathways, displayed as in **e**

To identify genes with altered expression during aging, we used DESeq2 [31] to calculate the fold-change in expression of each gene between the young, old, and geriatric dogs (Methods). To address the unbalanced sex distribution across age groups (Table 1), we included sex as a covariate in all differential expression models to control for potential confounding effects. The combined comparison of aged (old and geriatric, *n* = 30) dogs compared to young dogs (*n* = 10), revealed 816 differentially expressed genes at a false-discovery rate (FDR) of 10% (Fig. 2a,Supplementary Table S1). Separate analyses of geriatric versus young dogs and old versus young dogs identified 3702 and 8 differentially expressed genes, respectively (Fig. 2b, c), indicating that transcriptomic distinctions are more pronounced between groups with greater age differences. Overall, these alterations formed a continuous pattern of age-related change, demonstrated by the strong correlation between the fold change values in old versus young and geriatric versus young comparisons (Pearson *r* = 0.64, *p* < 10^–100^, Fig. 2d).

To determine whether these age-associated gene expression changes affected genes at random or were enriched in specific biological pathways, we performed gene-set enrichment analysis (GSEA [49]). We tested whether the age-related genes identified in the aged (old and geriatric) versus young comparison were more likely to occur in specific biological pathways than would be expected by chance by comparing this gene set against curated pathway databases (Methods). These analyses revealed that 335 Gene Ontology (GO [3]) Biological Process pathways were significantly down-regulated with age, while 349 GO pathways were up-regulated (Fig. 2e, Supplementary Table S2). Similarly, examination of Reactome [12] pathways indicated that 304 pathways were down-regulated and 220 pathways were up-regulated in aged dogs (Fig. 2f,Supplementary Table S2). Among the upregulated pathways, several were indicative of fibrotic response and chronic inflammation characteristic of inflammaging, including cytokine signaling, interleukin signaling, and neutrophil degranulation. The formation of fibrotic deposits was further supported by altered expression of matrix metalloproteinases (MMPs) and collagen species, key regulators of extracellular matrix remodeling that mediate age-related tissue fibrosis and contribute to the inflammatory milieu. These results suggest that the transcriptomic differences observed between young and aged dogs are concentrated in particular biological processes, reinforcing the idea that aging involves coordinated changes in key pathways likely to be conserved across species.

### Age-related changes in the plasma proteome

**Fig. 3:**
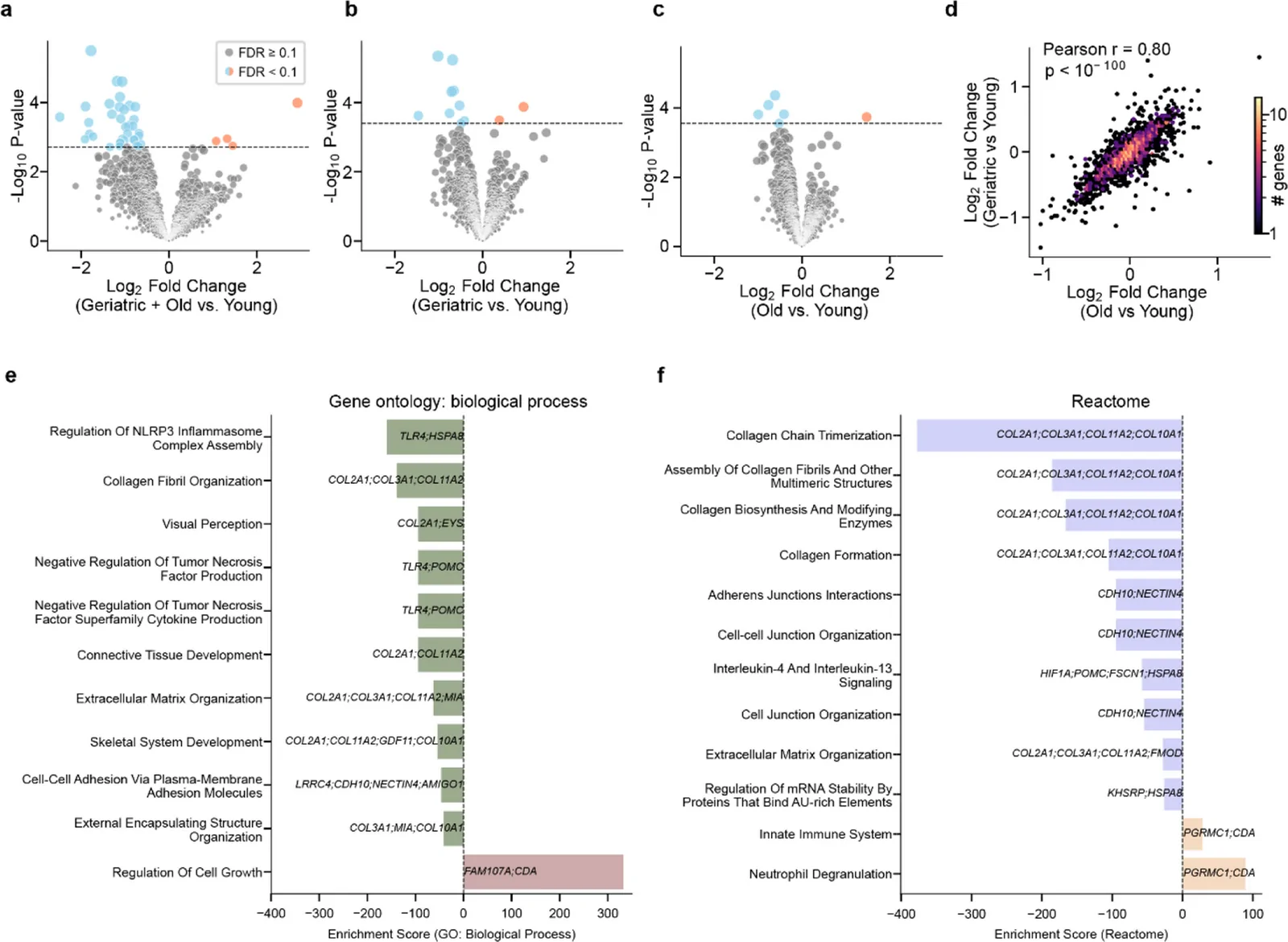
Proteomic alterations associated with aging in dogs. **a–c** Volcano plots depicting log_2_ fold change (*x*-axis) versus −log_10_ *p*-value (*y*-axis) for high confidence plasma proteins (*n* = 2,041 proteins) across three comparisons: **a** geriatric and old vs. young dogs, **b** geriatric vs. young dogs, and **c** old vs. young dogs. Proteins with FDR-corrected *p*-values < 0.1 (blue/orange) are considered significantly altered, whereas those with FDR-corrected *p*-values ≥ 0.1 (gray) are not (Methods). Significance was assessed through moderated t-statistics followed by false discovery rate adjustment in Limma. **d** Scatter plot illustrating the log_2_ fold change of each protein in geriatric vs. young (*y*-axis) and old vs. young (*x*-axis) comparisons. Points are colored by their log-scaled density. **e** Gene-set enrichment analysis (GSEA) results based on GO pathways. The ten most significantly up- and downregulated pathways are presented. Bar length (*x*-axis) indicates the enrichment score for genes downregulated (blue) or upregulated (red) with age in geriatric and old vs. young dogs (*n* = 30 dogs). **f** GSEA results for Reactome pathways, displayed as in **e**

In parallel to our transcriptomic analysis, we profiled the abundance of 6415 plasma proteins in the same beagle cohort (Methods). This proteomics assay is optimized for human proteins; thus, we focused our analysis on 2,041 proteins (“high-quality” set) determined by a prior study to be accurately quantified in dogs [48]. Consistent with the RNA-seq results, significant age-related changes in protein abundance were observed among all age groups; however, the number of differentially abundant proteins was markedly lower than the number of differentially expressed genes (*n* geriatric and old vs. young = 40 proteins, geriatric vs. young = 11 proteins, old vs. young = 6 proteins, Fig. 3a, c,Supplementary Table S3). Consistent with a gradual progression of molecular changes during aging, the fold changes in protein abundance between old and young dogs strongly correlated with those between geriatric and young dogs (Pearson *r* = 0.80, *p* < 10^–100^, Fig. 3d).

We next used GSEA to determine whether these proteomic alterations in aged dogs were enriched within specific biological pathways. In total, we found 46 down- and 1 up-regulated pathways in the GO database (Fig. 3e, Supplementary Table S4) and 18 down- and 2 upregulated pathways in the Reactome database (Fig. 3f, Supplementary Table S4).

We observed highly similar results between the “high-quality” set of proteins and all detected proteins (*n* = 6415 proteins, Supplementary Fig. 1a–f), with 39, 25, and 6 differentially abundant proteins observed between aged (old and geriatric) and young, geriatric and young, and old and young dogs, respectively.

### Concordance between dog and human aging

**Fig. 4:**
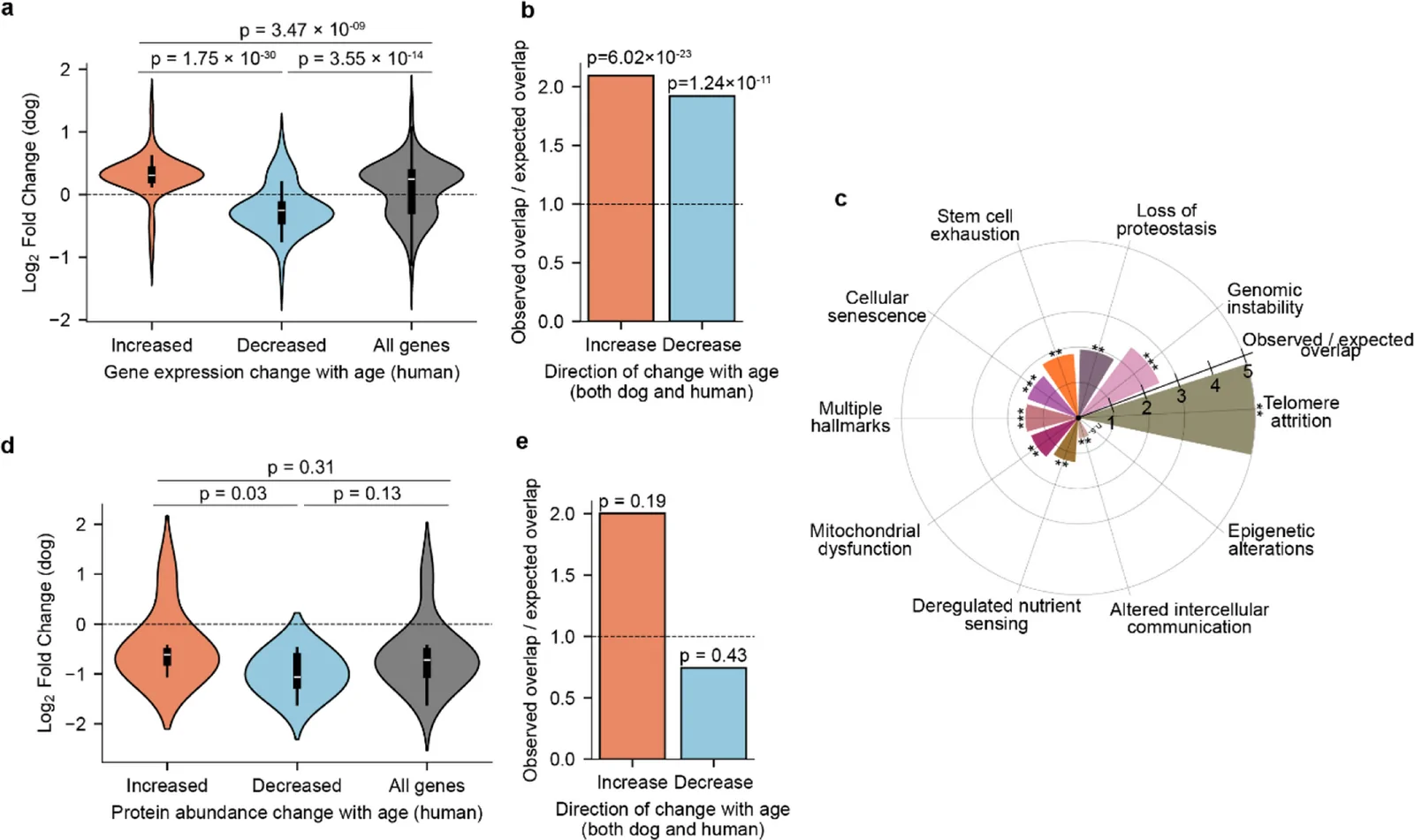
Comparison between molecular changes in dogs and humans with age. **a** Violin plot depicting the distribution of log_2_ fold-change values in geriatric and old vs. young dogs for genes profiled in this study and the human gene expression study [40] (*n* = 9827 genes). Genes are grouped by whether they significantly increase with age in humans (red), decrease with age in humans (blue), or represent the background distribution of all detected genes (gray). Significance was assessed using two-sided Mann–Whitney *U* tests. **b** Bar plot illustrating observed vs. expected overlap of genes that either increase (red bar, *n* = 220 genes) or decrease (blue bar, *n* = 130 genes) with age both in dogs and humans. Significance was assessed using a two-sided binomial test. **c** Radar plot showing the observed vs. expected overlap between genes significantly altered with age in dogs (geriatric and old vs. young dogs, *n* = 816 genes) and those annotated to specific hallmarks of aging (*n* = 413 genes, “Methods”). Bar height represents the degree of enrichment, with (***) and (**) denoting p-values of < 5.0 × 10⁻^14^ and < 5.0 × 10⁻⁸, respectively, from two-sided binomial tests. **d** Violin plot, as in **a**, depicting the distribution of log_2_ fold-change values for proteins in geriatric and old vs. young dogs stratified by whether these proteins increase (red), decrease (blue), or are part of the background set (gray) in the human protein study [46] (*n* = 513 proteins). **e** Bar plot analogous to **b**, showing the observed vs. expected overlap of proteins increasing (red, *n* = 3 proteins) or decreasing (blue, *n* = 11 proteins) with age in dogs vs. humans

To evaluate the extent of conservation in aging-related molecular alterations between dogs and humans, we compared the differentially expressed genes identified in aged (old and geriatric) vs. young dogs to those reported in a large-scale transcriptomic study of human aging [40]. As both datasets were derived from whole blood, this comparison enabled a direct cross-species evaluation. We observed a significant overlap in both up- and downregulated genes between species (Fig. 4a). When compared to random chance, we found enrichments of 2.1-fold for upregulated genes and 1.9-fold for downregulated genes (Fig. 4b). Furthermore, the age-associated transcriptomic changes in dogs were markedly enriched for genes related to the human hallmarks of aging (Fig. 4c, Methods). Specifically, there was a 5.0-fold increased likelihood for genes related to telomere attrition to change in expression with age in dogs (e.g., *TERT*), a 2.5-fold increase for genes implicated in genomic instability (e.g., *CDKN1A*,* CHEK2*, and *RFC4*), and a 1.9-fold increase for genes linked to the loss of proteostasis (e.g., *DBN1*, *TIMP1*, and *HSPA1A*). Of all the hallmarks of aging, epigenetic alterations were the only hallmark for which the age-related genes detected in this cohort did not exhibit significant overlap with (*p* = 1.0). These enrichments underscore the central role of these pathways in the aging process and reinforce the shared biology of aging between dogs and humans.

In contrast, when we compared the age-related plasma proteins in dogs with two datasets of proteomic alterations seen during human aging [37, 46], we found no significant overlap (Fig. 4d, We, Supplementary Fig. 2a-b). This discrepancy may reflect true species-specific differences in protein regulation. Alternatively, this dissimilarity may be due to methodological variations across studies or differential detection of canine versus human orthologs by the SomaScan assay.

Together, these findings suggest that while transcriptomic signatures of aging are well-conserved between dogs and humans, proteomic alterations may be more species-specific or governed by distinct regulatory mechanisms.

## Discussion

In this study, we have catalogued the extensive transcriptomic and proteomic alterations that occur during canine aging. Our findings reveal notable similarities in the molecular signatures of aging between dogs and humans. These findings emphasize the value of dogs as a translational model for human research and, reciprocally, the relevance of results from human aging studies in informing canine aging interventions. To support further research into the biology of aging across species, we have made these data publicly available at https://loyal-for-stats.shinyapps.io/beagle-omics/.

The biological pathways enriched for age-related changes in our transcriptomic and proteomic analyses mirror several well-established aging hallmarks, reinforcing their conservation across species. In both the transcriptome and proteome, we observed significant increases in the expression of inflammatory pathways with age, marked by an up-regulation of cytokine and interleukin signalling, neutrophil degranulation, and the innate immune system broadly (Figs. 2 and 3). These changes resonate with prior reports in both humans and model organisms, where pro-inflammatory shifts—termed “inflammaging”—have been linked to aging [16] and disease [15]. In particular, these results support a shift of the immune milieu toward myeloid activation and away from counter-regulatory Type-2 cues—as demonstrated by the elevation of neutrophil degranulation and phagocytosis/endocytosis while IL-4/IL-13 signaling is diminished—consistent with prior reports of age-related myeloid skewing [43]. In the transcriptome, DNA replication and repair programs were broadly diminished—encompassing homologous recombination and replication-fork maintenance genes—echoing reports across mammalian tissues that aging associates with reduced DNA repair capacity [30]. The proteome revealed marked reductions in extracellular matrix biosynthesis and assembly—collagens and processing components (*COL2A1*,* COL3A1*, and* COL10A1*) and junctional proteins (*CDH10*,* NECTIN4*)—consistent with established age-associated ECM decline and barrier weakening in humans [17]. This upregulation of inflammatory factors and down-regulation of the cell-cycle reflect alterations characteristic of senescent cells, the accumulation of which is another hallmark of aging [30]. The great extent to which the molecular changes arising during aging in dogs recapitulate aging hallmarks discovered in other species underscores the evolutionarily conserved nature of the aging process.

While all species must contend with universal biological constraints and environmental pressures—driving the evolution of core stress response and maintenance pathways like DNA repair, proteostasis, and nutrient sensing [30]—the long history of cohabitation between dogs and humans has resulted in a particularly close alignment in their aging processes [21, 45]. Genetic evidence suggests that dogs and humans have cohabitated for at least 23,000 years [39], providing sufficient time for genetic adaptations to have emerged, such as an enhanced ability by dogs to metabolize starch-heavy, human foods [4]. Consistent with these evolutionary and environmental commonalities, our transcriptomic analysis revealed a striking overlap between the age-related changes observed in dogs and those reported in human studies (Fig. 4). In contrast, this overlap was not reflected in the proteomic data, potentially indicating differences between dog and human aging at the post-translational level (Fig. 4, Supplementary Fig. 2). However, technical limitations likely also play a role: an altered affinity of the SomaScan 7 k assay for canine versus human proteins has been reported, with only ~ 29% of the measured proteins exhibiting highly confident quantification in dogs [48]. Nevertheless, pathway‑level analyses of the proteome recovered multiple canonical hallmarks of aging (Fig. 3), emphasizing the deep conservation of molecular aging programs and reinforcing the utility of the dog in translational geroscience.

Our study has several limitations that warrant consideration. First, although our dataset provides a valuable initial look at the molecular hallmarks of aging in dogs, it is constrained by its relatively small size (*n* = 40 dogs). Second, while our decision to focus on a single breed enabled us to reduce genetic heterogeneity and detect clearer signals of molecular change, it also limits the generalizability of our findings to other dog breeds or companion dogs living outside of a laboratory setting. However, recent literature has begun to describe the conserved parallels in aging-related metabolic and immunological changes between laboratory beagles and diverse real-world populations of companion dogs [33]. Third, further investigation is necessary to determine to what extent the differential detection of canine and human proteins by the SomaScan assay was a cause of the limited overlap between age-associated proteins in each species. Fourth, our analysis was limited to measurements in the blood. While our examination of the aging blood transcriptome delineates the gene expression changes occurring within white blood cells during aging, the capacity to extrapolate these findings to other tissues is limited. On the other hand, plasma proteomics offers a window into systemic changes affecting multiple organ systems [37], but it still does not capture tissue-specific nuances as thoroughly as direct tissue sampling. Fifth, while both lab beagles and companion dogs are part of *Canis lupus familiaris*, there can be differences between these groups underpinned by physiology and environmental factors. Therefore, further research on biomarkers of aging in companion dogs is necessary to ensure the results presented here are not particular to laboratory beagles. Additionally, the unbalanced sampling across sex and age groups induced confounding between these variables. While we addressed this limitation by including sex as a covariate in our statistical models, future studies would benefit from balanced sampling designs to more robustly disentangle age and sex effects. Finally, our cross-sectional design does not allow us to make definitive causal inferences about the biological mechanisms driving aging-related changes. Future studies with larger cohorts, balanced sex distributions, multiple breeds, and longitudinal sampling across diverse tissues will help validate and expand upon the molecular signatures of canine aging identified here.

## Methods

### Animal cohort

Forty gonadectomized male and female laboratory beagles weighing 8.5 to 14.0 kg were included in the study. Animals were included if they were in good general health, as determined by historical veterinary examination records and historical hematology and clinical chemistry results. Animals were excluded if they were pregnant or lactating, or if records indicated any underlying medical conditions. The dogs were stratified into three groups determined by their age. The “Young” group consisted of 10 beagles ranging from 3.3 to 4.7 years old, the “Old” group consisted of 17 beagles ranging from 8.0 to 9.2 years old, and the “Geriatric” group consisted of 13 beagles ranging from 10.5 to 13.7 years old. No dogs were excluded on the basis of age; gaps in the age range between groups (e.g., 5 to 6 years old) are due to the limited age diversity present in the cohort. Dogs were group housed in pens with access to environmental enrichment. The animal housing room maintained a 12 h light–dark cycle and temperatures within 15 °C to 28 °C. Dogs were fed a standard commercial diet (Purina^Ⓡ^ ProPlan^Ⓡ^ All Ages Sport Active 27/17 Chicken & Rice Formula) and were provided water ad libitum. The study is reported in accordance with ARRIVE guidelines.

Blood collections for endpoint analysis were completed in a fasted state, a minimum of 8 h from the previous feeding. The protocol was approved by the institutional animal care and use committee (IACUC; IVS218-22027-DZ). Blood was collected via jugular venipuncture to assess intracellular RNA and plasma proteins. For intracellular RNA samples, whole blood was transferred into PAXgene^Ⓡ^ RNA tubes (BD Biosciences) and stored at −20 °C until ready for shipment to the analytical site. For plasma protein samples, whole blood was transferred to K₂EDTA tubes and placed on wet ice until centrifuged at 2800–3200 RPM for 10 min at 4 °C. The resultant plasma was transferred to cryovials and frozen at −80 °C until ready for shipment to the analytical site.

### RNA extraction, library preparation with poly(A) selection and Illumina sequencing

Total RNA was extracted using the PAXgene Blood RNA Kit following the manufacturer’s instructions (Qiagen, Hilden, Germany). Post extraction, total RNA samples were quantified using the Qubit 2.0 Fluorometer (Life Technologies, Carlsbad, CA, USA), and RNA integrity was checked using the Agilent TapeStation 4200 (Agilent Technologies, Palo Alto, CA, USA). Globin depletion was performed using the QIAGEN FastSelect Globin depletion kit (Qiagen, Germantown, MD, USA). RNA sequencing libraries were prepared using the NEBNext Ultra II RNA Library Prep Kit for Illumina following the manufacturer’s instructions (NEB, Ipswich, MA, USA). Briefly, mRNAs were initially enriched with Oligod(T) beads. Enriched mRNAs were fragmented for 15 min at 94 °C. First strand and second strand cDNA were subsequently synthesized. cDNA fragments were end repaired and adenylated at 3′ ends, and universal adapters were ligated to cDNA fragments, followed by index addition and library enrichment by PCR with limited cycles. The sequencing library was validated on the Agilent TapeStation (Agilent Technologies, Palo Alto, CA, USA) and quantified by using the Qubit 2.0 Fluorometer (Invitrogen, Carlsbad, CA) as well as by quantitative PCR (KAPA Biosystems, Wilmington, MA, USA). The sequencing libraries were multiplexed and clustered onto a flow cell on the Illumina NovaSeq instrument according to the manufacturer’s instructions. The samples were sequenced using a 2 × 150 bp Paired End (PE) configuration. Image analysis and base calling were conducted by the NovaSeq Control Software (NCS). Raw sequence data (.bcl files) generated from the Illumina NovaSeq was converted into fastq files and de-multiplexed using the Illumina bcl2fastq 2.20 software. One mismatch was allowed for index sequence identification.

### Protein abundance detection protocol

Plasma proteins were profiled using the SomaScan® Assay (SomaLogic, Inc.), a high-throughput, aptamer-based proteomic platform capable of quantifying over 7,000 proteins from a single sample. Briefly, the SomaScan Assay leverages proprietary Slow Off-rate Modified Aptamers (SOMAmer® reagents)—chemically modified oligonucleotides that adopt complex three-dimensional structures and bind to specific epitopes of target proteins with high affinity and specificity. While highly optimized for human samples, this assay is less optimized for the orthologous proteins in dogs. Alternative protein quantification platforms, including Olink, were considered but presented comparable limitations in cross-species applicability. SomaScan was ultimately selected given its extensive validation in human aging studies [37] and the lack of available canine reference proteomes required for unbiased mass spectrometry-based approaches.

### Sample preparation and binding

Plasma samples were collected in K₂EDTA tubes, centrifuged at 2800–3200 g for 10 min at 4 °C, and the supernatant was stored at −80 °C until use. Each plasma sample was thawed on ice immediately prior to assay setup. SOMAmer reagents, pre-immobilized on streptavidin-coated beads via a Cyanine-3 fluorophore and a photocleavable biotin linker, were incubated with the plasma samples in the presence of proprietary buffers.

### Washing, biotinylation, and photocleavage

After incubation, unbound proteins were removed by a series of stringent washes, and bound proteins were then covalently biotinylated using NHS-biotin. After further washes to remove any free proteins, the SOMAmer–protein complexes were denatured with a chaotropic salt (sodium perchlorate), releasing both protein and SOMAmer aptamer.

### Microarray hybridization and detection

The liberated SOMAmer reagents were then hybridized overnight onto a custom Agilent microarray containing probes complementary to each SOMAmer. After stringent washing, the arrays were scanned on an Agilent microarray scanner to measure the Cy3 fluorescence intensity emitted by each feature.

### Proteomic quality control, calibration, and normalization

Raw Relative Fluorescence Units (RFU) values were used to compute plate-level calibration factors based on control wells containing calibration, quality control, and blank samples. For each SOMAmer reagent, the median RFU from the calibrator wells on a given plate was compared to the historical calibrator reference. The distribution of these SOMAmer-specific ratios was used to derive (i) a plate-wide scaling factor (the median ratio) and (ii) individual SOMAmer-specific calibration scale factors. Combined, these factors are corrected for plate-to-plate variation. Since canine proteins can exhibit sequence or structural differences from their human orthologs, an additional normalization step was performed using a study-specific reference pool generated from the canine plasma samples themselves. Each sample’s RFU profile was scaled relative to this pooled reference to account for any non-human binding differences. QC accuracy on each plate was subsequently confirmed by comparing the plate-specific QC ratios (QC well RFU relative to a historical QC reference) to predetermined acceptance criteria.

### RNA-seq quality control and processing

RNA-seq read processing was carried out using a Snakemake-based pipeline with tools and parameters as recommended by a recent review of RNA-seq processing methods [10]. Briefly, paired-end reads were quality- and adapter-trimmed using Trimmomatic (parameters: ILLUMINACLIP:TruSeq3-PE.fa:2:30:10, LEADING:3, TRAILING:3, SLIDINGWINDOW:4:15, MINLEN:36), producing paired and unpaired output FASTQ files. Following trimming, reads were aligned to the Canis lupus familiaris reference genome (ROS_Cfam_1.0) using STAR. For alignment, standard parameters were applied, including the use of the vendor-provided STAR index and annotation file. Aligned reads were sorted by genomic coordinates, and read counts per gene were computed with HTSeq (version 0.11.2; specifying -f bam -r name -s no -t exon -i gene_id -m union). These read counts were used in subsequent differential expression and downstream analyses.

### Detection of differentially expressed genes

The PyDESeq2 [35] implementation of the DESeq2 [31] algorithm was applied to identify age-related genes in the RNA-seq data (*n* = 16,273 genes. DESeq2 estimates size factors internally to normalize for library size, fits a negative-binomial GLM for each gene (dispersion estimation, trend fitting and empirical Bayes shrinkage, and performs Wald tests for specified contrasts. Three age contrasts were tested (old vs young, geriatric vs young, and aged [old and geriatric] vs young as well as the main effect of sex, all with Cook’s distance outlier filtering and independent filtering enabled. Age was analyzed categorically rather than as a continuous variable due to the discontinuous distribution of ages in our cohort [18], with distinct clusters of young (3.3–4.7 years), old (8.0–9.2 years), and geriatric (10.5–13.7 years) dogs and substantial gaps between these groups (Fig. 1c). Accidental imbalance in sampling across sex induced confounding between sex and age, and therefore, sex was included as a covariate in these models to account for this sampling bias. Weight was not included as a covariate because it was strongly collinear with sex (males = 12.38 ± 0.80 kg, females = 10.22 ± 1.01 kg), and simultaneous inclusion would result in multicollinearity, a known statistical problem that leads to inflated standard errors and biased parameter estimation [13]. Weight did not vary significantly with age in our cohort (Supplementary Fig. 3a-b)**,** indicating that weight is not a confounder of age-related changes in expression levels and does not require adjustment. Additional sensitivity analyses confirmed that inclusion of weight alone (and not sex) produced nearly identical results (data not shown). Thus, because sex is the underlying biological factor influencing weight, but not vice versa, we adjusted for sex rather than weight. Raw p-values were adjusted by the Benjamini-Hochberg [7] method to control the false discovery rate at 10%. Genes with FDR < 0.10 were deemed differentially expressed.

### Detection of differentially expressed proteins

The Inmoose [9] port of the Limma package [42] was used to identify age‐associated proteins in the SomaScan 7 K data. First, the relative fluorescent units (RFU) matrix was log₂‐transformed. Next, a linear model including age group (Young, Old, and Geriatric) and sex (male, female) as covariates was fit using lmFit, followed by empirical Bayes moderation (eBayes). Finally, three age contrasts were evaluated (old vs young, geriatric vs young, and combined aged [old and geriatric] vs young) with re-moderation. As in the analysis of transcriptomic data, age was analyzed categorically and sex was adjusted for. Raw p-values were adjusted by the Benjamini–Hochberg method to control the false discovery rate at 10%, and proteins with FDR < 0.10 were deemed differentially abundant. This process was carried out on all proteins (*n* = 6415 proteins) as well as the set of proteins determined by an internal SomaLogic quality assessment to be “high confidence” in their detection (*n* = 2041 proteins) [48].

### Enrichment analyses

Gene set enrichment analysis was conducted using a custom Python workflow that leveraged the Enrichr interface of the gseapy [14] library. Briefly, significantly up- or down-regulated genes (FDR < 0.1) were provided to the enrichment function along with a relevant background set encompassing all measured transcripts. Analysis was carried out separately for Gene Ontology [3] and Reactome [12] pathways. Pathways with a *p*-value < 0.1 were retained, and the top ten terms by fold-change were plotted with up to five genes within each enriched pathway annotated next to the corresponding bars (in order of gene fold-change in expression).

### Comparison with molecular aging in humans

Whether aging-related alterations in dogs overlapped with those observed in humans was assessed by comparing differentially expressed canine genes or proteins against published human aging datasets. Dog genes were mapped to their human orthologs using the python implementation (pyBiomart) of Biomart [47]. For transcriptomic comparisons, publicly available whole-blood RNA-seq data from Peters et al. [40] were used, in which genes were classified as either increasing, decreasing, or not significantly changing with age. Comparison of gene expression in aged (old and geriatric) versus young dogs was used to categorize genes in dogs accordingly. Then, the number of canine genes showing the same directional change as humans (e.g., increased in both dogs and humans) was tallied. The expected overlap between these sets was calculated as the proportion of genes significantly increased/decreased with age in the dog dataset multiplied by the proportion of genes significantly increased/decreased with age in the human dataset, scaled by the total number of genes tested. A two-sided binomial test was used to determine whether the observed intersection differed significantly from this expected number of overlapping genes. Enrichment ratios were computed as the observed overlap divided by the expected overlap. Analogous steps were taken for comparing age-related protein changes in dogs with two independent human plasma proteomic datasets [37, 46].

### Overlap of canine age-related genes with the hallmarks of aging

The overlap between the canine genes altered in expression with age and genes implicated in the hallmarks of aging was assessed. Genes implicated in the hallmarks of aging were obtained from the National Genomics Data Center (NGDC) Aging Atlas (https://ngdc.cncb.ac.cn/aging/age_related_genes). From this dataset, only hallmark categories comprising at least 20 measured canine genes were retained. Two non-hallmark categories, “NF-κB related gene” and “senescence-associated secretory phenotype,” were excluded, and the “others” category (composed of multiple overlapping hallmarks) was renamed to “Multiple hallmarks.” Next, canine genes were mapped to their human orthologs using Biomart [47] and the hallmark genes were intersected with the set of age-related genes found in the comparison of gene expression between aged (old and geriatric) versus young dogs. The observed overlap between this subset and each hallmark category was computed and compared to the expected overlap under random assumptions. The expected overlap at random was calculated by multiplying two proportions: (i) the fraction of all measured dog genes that were age-related and (ii) the fraction belonging to each hallmark category. Then, the enrichment of age-related dog genes for a given hallmark was computed by dividing the observed proportion of hallmark-associated genes that were age-related in dogs by the expected proportion. A two-sided binomial test was used to assess whether the observed overlap exceeded the expected overlap for each hallmark category.

## Supplementary Information

Below is the link to the electronic supplementary material.

ESM1 (ZIP 9.39 MB)
