# Supplementary material for: Multi-omic analysis of canine aging uncovers conserved aging pathways
Source: GeroScience. 2025 Dec 17;48(5):7215–28. doi: 10.1007/s11357-025-02029-2 (PMC13601455; doi:10.1007/s11357-025-02029-2)
Supplement: Supplementary file 1 [file 11357_2025_2029_MOESM1_ESM.zip › supplementary_figures.docx]

## Supplementary figures

### **Supplementary figure 1: Supplementary volcano plots**

**a–c)** Volcano plots depicting log_2_ fold change (x-axis) versus –log_10_ p-value (y-axis) for all detected plasma proteins (n = 6,415 proteins) across three comparisons: (a) geriatric and old vs. young dogs, (b) geriatric vs. young dogs, and (c) old vs. young dogs. Proteins with FDR-corrected p-values < 0.1 (blue/orange) are considered significantly altered, whereas those with FDR-corrected p-values ≥ 0.1 (gray) are not (**Methods**). Significance was assessed through moderated t-statistics followed by false discovery rate adjustment in Limma.

**d)** Scatter plot illustrating the log_2_ fold change of each protein in geriatric vs. young (y-axis) and old vs. young (x-axis) comparisons. Points are colored by their log-scaled density.

**e)** Gene-set enrichment analysis (GSEA) results based on GO pathways. The ten most significantly up- and down-regulated pathways are presented. Bar length (x-axis) indicates the enrichment score for genes downregulated (blue) or upregulated (red) with age in geriatric and old vs. young dogs (n=30 dogs).

**f)** GSEA results for Reactome pathways, displayed as in (e).

### **Supplementary figure 2: Lack of overlap between proteomic changes in humans vs. dogs**

**(a)** Violin plot depicting the distribution of log_2_ fold-change values for proteins in geriatric and old vs. young dogs (n = 1,445 proteins). Proteins are grouped by whether they significantly increase with age in humans (red), decrease with age in humans (blue), or represent the background distribution of all detected proteins (gray) in the human protein abundance study [(Oh et al. 2023)](https://paperpile.com/c/p1Db1F/3Boa). Significance was assessed using two-sided Mann–Whitney U tests.

**(b)** Bar plot illustrating observed vs. expected overlap of proteins that either increase (red bar, n = 8 proteins) or decrease (blue bar, n = 57 proteins) with age both dogs and humans. Significance was assessed using two-sided binomial tests.

### **Supplementary Figure 3: Relationship between age and weight**

**a)** Scatter plot depicting the relationship between age (x-axis) and body weight (y-axis) across all dogs (n = 40). The line of best fit (green) with 95% confidence interval (shaded region) shows no significant correlation between age and weight (Spearman ρ = -0.24, p = 0.14).

**b)** Scatter plot of age versus body weight stratified by sex, with males (blue, n = 11) and females (orange, n = 29) shown separately. Lines of best fit with 95% confidence intervals demonstrate no significant age-weight correlation in either males (Spearman ρ = -0.10, p = 0.77) or females (Spearman ρ = 0.05, p = 0.80).
